# Single-cell analysis of oxidative phosphorylation protein expression in pancreatic islets in type 2 diabetes

**DOI:** 10.1530/JOE-25-0253

**Published:** 2025-10-23

**Authors:** Alana Mullins, Xuefei Yu, Anna L M Smith, George Merces, James A M Shaw, Laura C Greaves, Mark Walker, Catherine Arden

**Affiliations:** ^1^ Biosciences Institute, Newcastle University, Newcastle Upon Tyne, UK; ^2^ Department of Critical Care Medicine, Renji Hospital, School of Medicine, Shanghai Jiao Tong University, Shanghai, China; ^3^ Biosciences Institute, Innovation, Methodology and Application (IMA) Research Theme, Faculty of Medical Sciences, Newcastle University, Newcastle upon Tyne, UK; ^4^ Image Analysis Unit, Faculty of Medical Sciences, Newcastle University, Newcastle upon Tyne, UK; ^5^ Translational & Clinical Research Institute, Faculty of Medical Sciences, Newcastle University, Newcastle upon Tyne, UK; ^6^ School of Cancer Sciences, University of Glasgow, Glasgow, UK

**Keywords:** bi-hormonal cells, islet architecture, machine learning, mitochondrial dysfunction, oxidative phosphorylation, single-cell analysis and type 2 diabetes

## Abstract

Mitochondrial dysfunction is a key feature of type 2 diabetes and is closely linked to ageing, a major risk factor for the disease. This study investigated islet cell composition and mitochondrial oxidative phosphorylation protein expression in pancreatic tissue from older donors (≥62 years) with and without type 2 diabetes, matched for age, sex, and BMI. Fixed human pancreatic tissue sections were immunolabelled for insulin, glucagon, NDUFB8 (complex I), MTCO1 (complex IV), and VDAC1 (a mitochondrial mass marker) to quantify islet composition and mitochondrial protein levels. A machine learning-based single-cell segmentation pipeline enabled high-resolution profiling of individual cell populations within islets. In type 2 diabetes, islets exhibited an increased alpha:beta cell ratio, altered spatial organisation with fewer beta–beta and more alpha–alpha interactions, and a significantly higher proportion of bi-hormonal cells co-expressing insulin and glucagon. Within beta cells, we observed significant changes in mitochondrial protein expression, including reduced complex I and elevated complex IV levels. Unsupervised clustering of mitochondrial expression patterns identified three distinct beta cell expression clusters. Donors with type 2 diabetes showed a marked shift in the distribution of beta cells across clusters, with increased proportions of beta cells exhibiting low complex I and high complex IV expression. These results highlight significant alterations in islet architecture and mitochondrial protein expression associated with type 2 diabetes, providing new insights into the mechanisms underlying type 2 diabetes.

## Introduction

By 2035, the prevalence of type 2 diabetes in England is projected to reach 4.41 million people, accounting for 9.7% of the adult population (1). Saliently, there are marked disparities in type 2 diabetes prevalence across demographic groups, with older adults exhibiting significantly higher rates. Health data published by the National Health Service (NHS) in the UK indicate that 60% of all individuals diagnosed with type 2 diabetes in England are aged between 60 and 79 years, with a projected annual increase of 4.3% (2).

Ageing and type 2 diabetes share mitochondrial dysfunction as a common pathophysiological feature, exerting synergistic detrimental effects in older adults (3, 4). Mitochondrial dysfunction is central to the pathogenesis of type 2 diabetes, contributing to both insulin resistance and beta cell dysfunction (5, 6). Mitochondria play a critical role in regulating glucose-stimulated insulin secretion in pancreatic beta cells, with adenosine triphosphate (ATP) production serving as the metabolic link between blood glucose levels and insulin release (7). In individuals with type 2 diabetes, mitochondrial dysfunction is associated with increased oxidative stress and reactive oxygen species (ROS) production, dysregulated mitochondrial dynamics and mitophagy, impaired mitochondrial biogenesis, mitochondrial swelling, and reduced ATP levels (8, 9, 10, 11, 12, 13). The cumulative effects of these mitochondrial abnormalities ultimately result in progressive beta cell failure and attrition of cellular mass in type 2 diabetes.

Targeting mitochondrial dysfunction presents a promising therapeutic avenue for preserving beta cell function in type 2 diabetes. However, the molecular defects underlying mitochondrial dysfunction in human type 2 diabetes remain inadequately characterised. Insights from the *PolgA^mut/mut^* mouse model, which harbours a mutation in the polymerase proofreading domain of mitochondrial DNA (mtDNA) leading to accelerated accumulation of mtDNA mutations resulting in an advanced ageing phenotype, have revealed significant alterations in islet composition and the expression of mitochondrial oxidative phosphorylation (OXPHOS) complex proteins (14). Specifically, the *PolgA^mut/mut^* mouse model has demonstrated that at 44 weeks, mice exhibit alpha cell hyperplasia, reduced complex I expression, and increased complex IV expression in pancreatic beta cells, compared to wild-type controls. Similar alterations in islet endocrine cell proportions have been observed in human individuals with type 2 diabetes, with an increase in alpha:beta cell ratio (15, 16, 17, 18). However, despite these findings, there is a lack of established research on the corresponding changes in OXPHOS protein levels in human pancreatic islets. Prior human studies have reported that 21 OXPHOS genes (17 nuclear-encoded and 4 mitochondrial-encoded) are all downregulated in pancreatic islets from individuals with type 2 diabetes compared to controls without diabetes (19). Nonetheless, these transcriptomic findings have yet to be translated to an understanding of the corresponding protein expression profiles.

In the current study, we aimed to address this gap by conducting a phenotypic analysis of complex I and IV protein expression within human pancreatic islets. This was undertaken using fixed human pancreatic tissue from 14 deceased donors, comprising seven individuals with type 2 diabetes and seven without diabetes, all aged 62 years and over. To support our analysis at single-cell resolution, we developed a semi-automated image analysis pipeline, IsletAnalytics, using open-source tools and established machine learning-based segmentation. This pipeline was created specifically to answer our research questions, enabling the quantification of protein expression patterns within individual islet cells in the context of type 2 diabetes.

## Methods

### Human pancreas samples

Donors from the study were kindly provided by the Quality in Organ Donation Pancreas (QUOD-PANC) biobank, Newcastle (UK). The experimental cohort comprised seven donors without diabetes and seven donors with type 2 diabetes, all ≥62 years old and matched for age, sex, and body mass index (BMI) (Table 1). Additional clinical metadata were available for most parameters, including HbA1c levels for 4/7 T2D donors (range: 44–93 mmol/mol), diabetes duration for 5/7 T2D donors (range: <1–26 years), diabetes medication categories for all T2D donors (broadly classified as insulin +/− medication, diet, or medication without specific drug identification), and complete ischaemia time and comorbidity profiles for all 14 donors (Supplementary Table 1 (see section on Supplementary materials given at the end of the article)). Autoantibody status was not available for this donor cohort, limiting our ability to definitively exclude latent autoimmune diabetes in adults (LADA). All tissue sections were sourced from the body region of the pancreas. Work involving fixed human pancreatic tissue was conducted following ethical approval, and with the consent of the donor’s relatives, adhering to the Declaration of Helsinki.

**Table 1:** Clinical characteristics of 14 human donors used in this study. Donors are categorised by diabetes status, with seven donors with type 2 diabetes and seven without diabetes. Data include donor ID, age (in years), body mass index (BMI, in kg/m^2^), sex, diabetes status, anatomical region of pancreas, HbA1c (mmol/mol), diabetes medication categories (insulin +/− medication, diet, or medication without specific drug identification), duration since diabetes diagnosis, and the mean ± SEM for age and BMI by diabetes status.

Donor ID	Age (years)	BMI (kg/m^2^)	Sex	Diabetes status	Anatomical region of pancreas	HbA1c (mmol/mol)	Diabetes medication	Duration since diabetes diagnosis
Mean ± SEM (type 2 diabetes)	65.43 ± 1.36	32.13 ± 1.82						
Mean ± SEM (without diabetes)	67.0 ± 2.04	29.80 ± 1.01						
*P*-value	0.533	0.285						
PT0267_0020	70	34.9	Female	Type 2 diabetes	Body	52	Insulin+/−medication	Not reported
PT0267_0026	63	32.8	Female	Type 2 diabetes	Body	Not reported	Not reported	Not reported
PT0267_0051	71	28.6	Male	Type 2 diabetes	Body	44	Diet	<1 year
PT0267_0081	63	33.3	Male	Type 2 diabetes	Body	93	Insulin+/−medication	26 years
PT0267_0086	64	27.5	Female	Type 2 diabetes	Body	63	Insulin+/−medication	11 years
PT0267_0090	65	40.6	Female	Type 2 diabetes	Body	Not reported	Diet	22 years
PT0267_0095	62	27.2	Male	Type 2 diabetes	Body	Not reported	Medication	3 years
PT0267_0021	63	31.6	Female	Without diabetes	Body	NA	NA	NA
PT0267_0022	63	26.9	Male	Without diabetes	Body	NA	NA	NA
PT0267_0030	62	28.7	Male	Without diabetes	Body	NA	NA	NA
PT0267_0032	74	30.5	Male	Without diabetes	Body	NA	NA	NA
PT0267_0042	71	26.3	Female	Without diabetes	Body	NA	NA	NA
PT0267_0054	63	30.8	Female	Without diabetes	Body	NA	NA	NA
PT0267_0058	73	33.8	Female	Without diabetes	Body	NA	NA	NA

### Immunofluorescence

Four μm paraffin-embedded sections were deparaffinised in Histoclear™ and rehydrated through a graded ethanol series (100, 95, 70, 50% ethanol, then distilled water). Antigen retrieval was performed using sodium citrate (VWR Chemicals, 27833.294) (pH 6.0) for islet composition staining and Ethylenediaminetetraacetic acid (EDTA) (ThermoFisher, UK; J15700-A1) (pH 8.0) for mitochondrial protein labelling. Sections were blocked with 10% normal goat serum, followed by an Avidin/Biotin block (2BScientific, UK; SP-2001) for mitochondrial staining. Primary antibodies (voltage-dependent anion channel 1 (VDAC1), mitochondrial cytochrome oxidase I (MTCO1), NADH dehydrogenase [ubiquinone] 1 beta subcomplex subunit 8 (NDUFB8), insulin, glucagon, and E-cadherin) were incubated overnight at 4°C (Supplementary Table 1). Primary antibodies were applied as cocktails for each panel (islet composition panel: insulin + glucagon + E-cadherin; mitochondrial panel: NDUFB8 or MTCO1 + VDAC1 + insulin + E-cadherin). Each donor also had a sample in which no primary antibodies were added. The next day, sections were washed and incubated with Alexa Fluor-conjugated secondary antibodies for 2 h at room temperature (Supplementary Table 2). Islet composition slides were counterstained with 4′,6-diamidino-2-phenylindole (DAPI) (Merck, UK; D9542) for 15 min, and all slides were mounted using ProLong Gold Antifade Mountant (Thermo Fisher Scientific, UK; P36930).

### Image acquisition

Microscopic images were captured using a Zeiss LSM800 confocal laser scanning microscope equipped with 405, 488, 561, and 640 nm laser lines, along with two GaAsP detectors and one Airyscan detector, running ZEN Blue v3.10 software. A Plan Apo 20x/0.8 NA objective was used for image acquisition. For each donor, a low-magnification preview scan of the tissue was performed to identify islets containing >10 insulin-positive cells. From these eligible islets, up to 20 per donor were selected at random, provided they exhibited morphological integrity and adequate insulin staining, without bias toward specific architectural patterns. All samples were imaged using identical laser power and acquisition settings for each primary antibody across donors, with optimisation performed separately for each staining panel. All imaging was completed within 2 weeks of staining.

### IsletAnalytics analysis tool

**Figure 1:**
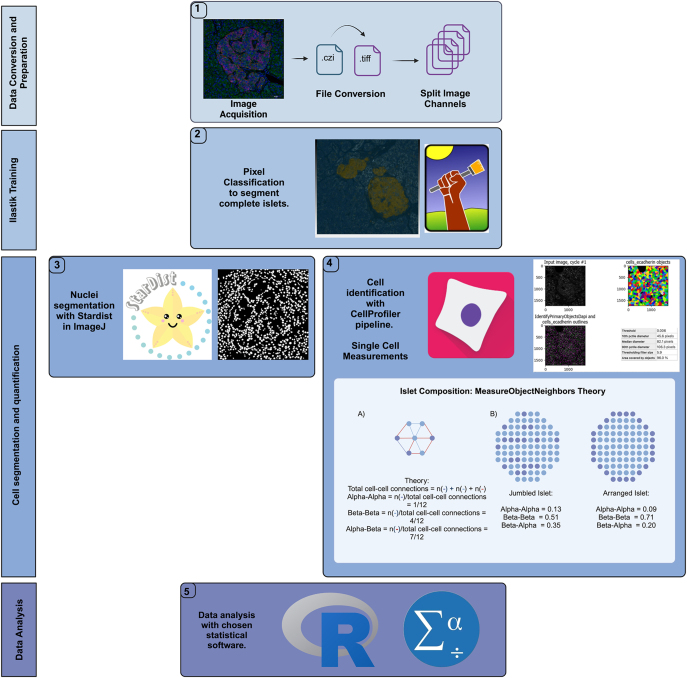
IsletAnalytics: semi-automated workflow for segmentation of immunofluorescent-stained human pancreatic islets. Data conversion and preparation (stage 1). i) Image acquisition: human pancreatic islet immunofluorescent images are acquired. File conversion: images are converted from czi to .tiff format. Channel splitting: images are processed to split the channels into their respective fluorophores. Iterative Ilastik training (stage 2). ii) Pixel classification: Ilastik’s random forest classifier is used for iterative training to segment complete islets through machine learning-based pixel classification. This approach addresses the challenge of islets in close proximity, enabling their individual analysis. Cell segmentation and quantification (stages 3 and 4). iii) Nuclei segmentation: nuclei within the islets are segmented using StarDist in ImageJ/Fiji. iv) Cell identification: cell segmentation is performed using a CellProfiler-designed pipeline, facilitating single-cell measurement. A schematic illustrates the MeasureObjectNeighbors module in CellProfiler, showing an islet composed of three alpha cells (slate blue) and four beta cells (light blue), with lines indicating contacts between neighbouring cells. In addition, it compares two architectural arrangements: a random mixture of cells (jumbled islet) on the left and a regular structure (arranged islet) on the right, depicting probabilities of contact between cell types based on arrangement. Data analysis (final stage). v) Statistical analysis: data analysis is conducted using the chosen statistical software. In this workflow, RStudio and SPSS are the preferred software for statistical analysis. A full-colour version of this figure is available at https://doi.org/10.1530/JOE-25-0253.

To quantify single cells within immunofluorescently labelled human pancreatic islets, we developed an open-source, semi-automated analysis pipeline incorporating established machine-learning tools (Fig. 1) (19, 20, 21, 22, 23). The pipeline combines established tools: Ilastik for supervised pixel classification, StarDist for nuclei segmentation, and CellProfiler for single-cell quantification. A comprehensive description is provided in Supplementary Document 1, and the pipeline’s code, along with R analysis scripts, is available via GitHub: https://github.com/amullins2/HumanIsletMLPipeline_2024.

### Statistical analysis

Data restructuring and visualisation were conducted in RStudio (v4.3.3). Cell counts for alpha and beta cells in islet composition and mitochondrial staining panels were determined with donor-specific thresholds. For mitochondrial protein analysis, OXPHOS protein quantification was performed exclusively on insulin-threshold-positive cells to ensure beta cell specificity, with alpha cell OXPHOS expression excluded from analysis. Donor-specific intensity thresholds for positive cell identification were determined by analysing the distribution of intensity values within each donor. Median and mean intensity values served as reference points, with thresholds set above these levels. Threshold determination was validated through qualitative assessment within CellProfiler to confirm accurate cell classification. This approach accommodated inter-donor variability in staining while ensuring internal consistency within each staining panel.

For islet composition analysis, alpha and beta cell counts were determined for each individual islet, from which alpha:beta cell ratios were calculated per islet. For mitochondrial cluster analysis, k-means clustering was performed separately for each OXPHOS protein using all individual beta cells across all donors and islets, with the optimal number of three clusters determined by elbow plots. However, cluster proportions for statistical comparison were calculated as the percentage of each donor's total beta cells (pooled across all islets from that donor) falling within each expression cluster.

Statistical analyses were performed using SPSS v29. Linear mixed models (LMMs) were used to analyse islet composition and mitochondrial data, incorporating sex, age, and BMI as covariates. For islet composition, fixed effects included sex, BMI, age, diabetes status, islet area, and cell connections, with random intercepts for donors and islets. Mitochondrial analysis employed a three-level hierarchical LMM with maximum likelihood estimation, with fixed effects including sex, BMI, age, diabetes status, and clusters, and a nested term between diabetes status and cluster. Residual plots were used to evaluate model fit. Comparisons were supported by Monte Carlo chi-squared and Bonferroni post-hoc analyses. Effect sizes were calculated using Cohen’s d for continuous variables and Cramer’s V for categorical variables to assess the magnitude of observed differences. Effect sizes were interpreted as small (Cohen’s d: 0.2, Cramer’s V: 0.1), medium (Cohen’s d: 0.5, Cramer’s V: 0.3), or large (Cohen’s d: 0.8, Cramer’s V: 0.5). Data are presented as mean ± standard error of the mean (SEM) for ratios and proportions (alpha:beta cell ratio, MTCO1/VDAC1 ratio, NDUFB8/VDAC1 ratio) and proportion ± SEM for bi-hormonal cells, cell connections, and expression clusters. A significance threshold of *P* < 0.05 was applied.

## Results

### IsletAnalytics analysis tool

**Figure 2:**
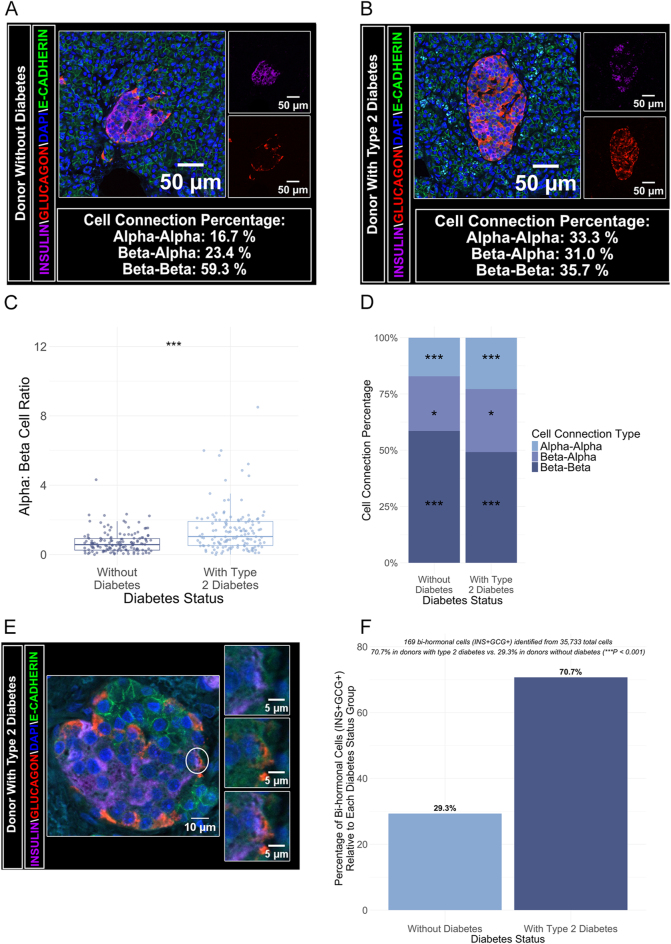
(A and B) Representative immunofluorescence images of pancreatic islets from matched donors: donor 5 (without diabetes, top row) and donor 6 (with type 2 diabetes, bottom row). Left panels show composite staining for DAPI (nuclear marker; Alexa Fluor 405), insulin (beta cell marker, Alexa Fluor 647), glucagon (alpha cell marker, Alexa Fluor 564), and E-cadherin (membrane marker; Alexa Fluor 488). Right panels display split channels for insulin and glucagon. Insets show the percentage of cell–cell interactions among alpha–alpha, beta-alpha, and beta–beta cell types. Scale bar = 50 μm. (C) Box plot comparing the alpha:beta cell ratio across *n* = 14 donors (seven without diabetes, seven with type 2 diabetes). Each box plot displays the median, interquartile range, and outliers, with individual islet data shown as jittered points. A linear mixed-effects model revealed a significant difference between groups (***P < 0.001). (D) Stacked bar chart illustrating the distribution of alpha–alpha, beta–alpha, and beta–beta cell–cell contacts by diabetes status across *n* = 14 donors. Percentages for each contact type are indicated in the legend: alpha–alpha (17.1% without diabetes, 22.8% with type 2 diabetes), beta–alpha (24.3 vs 28.0%), and beta–beta (58.6 vs 49.2%). A linear mixed-effects model showed significant differences in alpha–alpha (***P < 0.001), beta-alpha (*P < 0.05), and beta–beta (***P < 0.001) contact proportions. (E) Representative islet image from donor PT0267_0032 (type 2 diabetes) showing a bi-hormonal INS^+^GCG^+^ cell. The composite panel (left) includes a white circle marking the bi-hormonal cell. Right panels show individual channels for insulin, glucagon, DAPI, and E-cadherin used for segmentation, along with a magnified view of the marked cell. Scale bars: 10 μm for the composite image and 5 μm for the magnified and channel-specific images. (F) Bar chart showing the proportion of bi-hormonal cells (INS^+^GCG^+^) in donors without diabetes (29.3%) compared to those with type 2 diabetes (70.7%). Significance: *P < 0.05; **P < 0.01; ***P < 0.001. A full-colour version of this figure is available at https://doi.org/10.1530/JOE-25-0253.

We developed a customised image analysis pipeline designed to address specific research questions, leveraging established open-source software to enable single-cell resolution analysis of human pancreatic tissue (Fig. 1). We initially validated the pipeline by quantifying the alpha-to-beta cell ratio in individuals with type 2 diabetes (Fig. 2). Unlike conventional area-based quantification methods, our approach relies on threshold-based counts of individually segmented cells expressing key markers, such as insulin and glucagon. Single-cell segmentation is achieved using nuclear (DAPI) and membrane (E-cadherin) markers, allowing precise quantification of marker expression at the single-cell level.

### Increase in alpha:beta cell ratio in islets from donors with type 2 diabetes

Despite imaging the same number of islets in each cohort (Supplementary Fig. 1A), quantification of total cell numbers using threshold-positive labelling revealed significant differences in alpha and beta cell counts between donors with and without diabetes (Supplementary Fig. 1B and C). Donors without diabetes had a total alpha cell count of 6,134 and a beta cell count of 11,403, while donors with type 2 diabetes exhibited alpha and beta cell counts of 10,232 and 7,964, respectively. This reduction in total beta cell count in donors with type 2 diabetes highlights a substantial loss of beta cell mass at single-cell resolution. The alpha:beta cell ratio was calculated by determining the total alpha and beta cell counts per islet and calculating the ratio from these values. Donors with type 2 diabetes displayed a significantly higher alpha:beta cell ratio compared to those without diabetes (Figs. 2C and Supplementary Fig. 1D) (type 2 diabetes: 1.4 ± 0.11 vs without diabetes: 0.73 ± 0.053; ***P < 0.001, Cohen’s d = 1.979), consistent with previous reports in the literature (15, 16, 17, 18).

We further examined cell–cell interactions using CellProfiler’s MeasureObjectNeighbors module, focussing on beta–beta, alpha–alpha, and beta–alpha connections (Fig. 2A, B, D). In type 2 diabetes, the proportion of beta cells with direct beta–beta connections was significantly reduced compared to donors without diabetes (type 2 diabetes: 49.2% vs without diabetes: 58.6%, respectively; ***P < 0.001), while the proportion of alpha cells with direct alpha–alpha connections was significantly increased (type 2 diabetes: 22.8% vs without diabetes: 17.1%, respectively; ***P < 0.001). Despite these statistically significant differences, the overall alteration in islet architectural organisation remained modest, as indicated by a small effect size (Cramer’s V = 0.085). These findings collectively support the observed loss of beta cell mass, increased alpha:beta cell ratio, and altered cell–cell interactions in type 2 diabetes, which are conserved in older individuals with the disease.

### Greater number of bi-hormonal cells in islets from donors with type 2 diabetes

Bi-hormonal cells were classified based on the detection of both insulin (INS) and glucagon (GCG) signals within the same E-cadherin-defined cell boundary. From the analysis of 35,733 total cells, 169 (0.473%) were identified as threshold-positive bi-hormonal cells co-expressing insulin and glucagon (INS^+^GCG^+^) (Fig. 2E). Of these 169 INS^+^GCG^+^ cells, 70.7% were observed in donors with type 2 diabetes, while 29.3% were from donors without diabetes, representing a significantly higher proportion in type 2 diabetes (type 2 diabetes: 70.7% ± 0.5% vs without diabetes: 29.3% ± 0.4%, respectively; ***P < 0.001, Cramer’s V = 0.027) (Fig. 2F). Although the overall effect size was small (Cramer’s V = 0.027), this reflects the rarity of bi-hormonal cells within the total islet cell population. Nonetheless, their significantly greater prevalence in type 2 diabetes represents a substantial relative increase, consistent with the notion that bi-hormonal cells emerge as a hallmark of islet dysfunction (24).

### Protein expression analysis revealed a reduction in complex I and an increase in complex IV within beta cells of donors with type 2 diabetes

**Figure 3:**
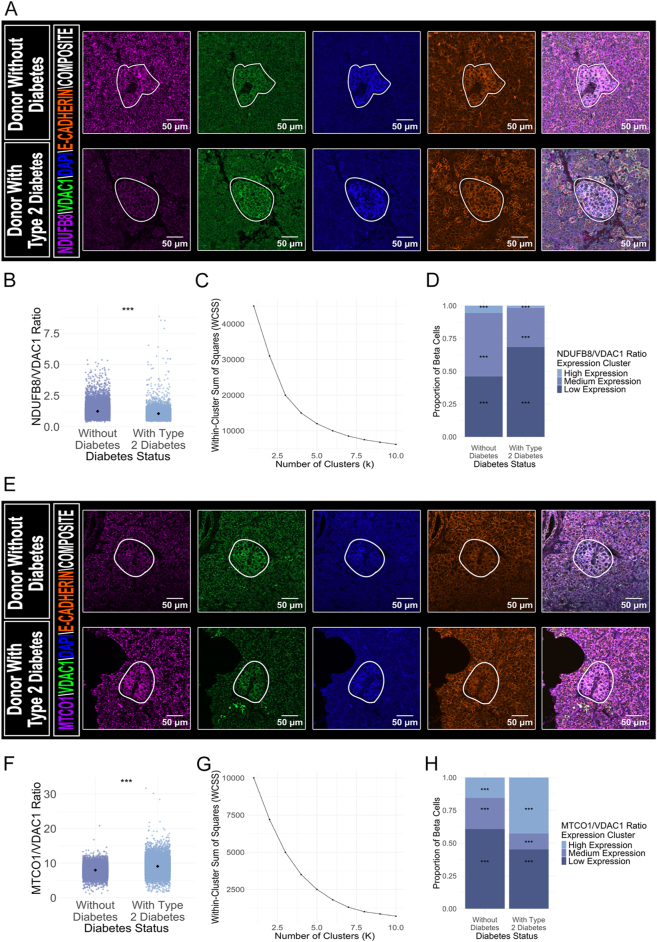
(A and E) Representative immunofluorescence images show pancreatic islets from donor 5 (without diabetes, top row) and donor 6 (with type 2 diabetes, bottom row), stained for NDUFB8 (complex I, Alexa Fluor 647), MTCO1 (complex IV, Alexa Fluor 647), VDAC1 (mitochondrial mass marker, Alexa Fluor 488), insulin (beta cell marker, Alexa Fluor 405), and E-cadherin (membrane marker, Alexa Fluor 546). White outlines demarcate islets selected for analysis. OXPHOS protein quantification was performed exclusively on insulin-threshold-positive cells following single-cell segmentation, ensuring beta cell specificity while excluding alpha cells to avoid confounding from altered alpha:beta cell ratios in type 2 diabetes. A reduction in the NDUFB8/VDAC1 ratio was observed in beta cells from donors with type 2 diabetes (A), while an increase in the MTCO1/VDAC1 ratio was seen in the same group (E). Scale bars represent 50 μm. (B and F) Jitter plots display NDUFB8/VDAC1 and MTCO1/VDAC1 ratios, respectively, for individual beta cells from 14 donors (seven without diabetes, seven with type 2 diabetes). Each point corresponds to a single beta cell, with black diamonds indicating group means. A linear mixed-effects model revealed significantly different mean expression ratios between groups (***P < 0.001). (C and G) Elbow plots illustrate the relationship between the number of clusters (x-axis) and the within-cluster sum of squares (y-axis) for the NDUFB8/VDAC1 and MTCO1/VDAC1 ratios, respectively. Clustering was performed using variables NDUFB8/VDAC1 or MTCO1/VDAC1 ratios, age, BMI, sex, and diabetes status. (D and H) Stacked bar charts display the proportions of beta cells categorised by intensity clusters of the NDUFB8/VDAC1 and MTCO1/VDAC1 ratios, respectively, based on data from 14 donors (seven without diabetes and seven with type 2 diabetes). ANOVA was used to validate the clustering results by comparing the means of expression across the three clusters. For the NDUFB8/VDAC1 ratio, significant differences were observed among clusters: low expression (0.907 ± 0.000965), medium expression (1.42 ± 0.00115), and high expression (2.30 ± 0.00682; all ***P < 0.001). For the MTCO1/VDAC1 ratio, the results were as follows: low expression (7.03 ± 0.0233), medium expression (7.74 ± 0.00996), and high expression (10.8 ± 0.0176; all ***P < 0.001). The proportions of beta cells in each cluster for the NDUFB8/VDAC1 ratio are as follows: low expression (dark blue; without diabetes: 46.0%, with type 2 diabetes: 68.4%), medium expression (blue; without diabetes: 48.4%, with type 2 diabetes: 29.8%), and high expression (light blue; without diabetes: 5.57%, with type 2 diabetes: 1.77%). For the MTCO1/VDAC1 ratio, the proportions are: low expression (dark blue; without diabetes: 60.7%, with type 2 diabetes: 45.2%), medium expression (blue; without diabetes: 23.7%, with type 2 diabetes: 12.0%), and high expression (light blue; without diabetes: 15.5%, with type 2 diabetes: 42.7%). A colour key for interpreting the clusters is provided in the plot legend. A LMM confirmed significant differences across clusters, and a Monte Carlo chi-squared test with Bonferroni post-hoc analysis further validated significant differences in proportions between all clusters (***P < 0.001). Significance: *P < 0.05; **P < 0.01; ***P < 0.001. A full-colour version of this figure is available at https://doi.org/10.1530/JOE-25-0253.

To assess mitochondrial function in human pancreatic tissue, we performed immunofluorescence staining for components of complex I (NDUFB8) and complex IV (MTCO1) (Fig. 3A and E). NDUFB8 and MTCO1 were analysed in separate immunofluorescence panels, precluding direct assessment of co-expression patterns within individual cells. Expression levels of these complexes were then normalised to mitochondrial mass using VDAC1 (25). Corresponding non-normalised values are presented in Supplementary Fig. 2A, B, C. No significant difference in VDAC1 expression was observed between donors with type 2 diabetes and their age-matched counterparts without diabetes (*P* = 0.461).

Normalised data (Fig. 3B and F) showed a significant reduction in complex I expression (NDUFB8/VDAC1 ratio: type 2 diabetes: 1.10 ± 0.00216 vs without diabetes: 1.28 ± 0.00346; ***P < 0.001, Cohen’s d = −0.490; *n* = 8,219 vs 11,743 cells) and an increase in complex IV expression (MTCO1/VDAC1 ratio: type 2 diabetes: 9.09 ± 0.0209 vs without diabetes: 7.99 ± 0.0132; ***P < 0.001, Cohen’s d = 0.600; *n* = 8,011 vs 11,947 cells) in beta cells from individuals with type 2 diabetes compared to those without diabetes. The heterogeneity observed in expression patterns led to a non-subjective categorisation of VDAC1-normalised OXPHOS expression using k-means clustering.

K-means clustering was utilised to categorise beta cells based on their individual protein expression profiles, with separate analyses for each OXPHOS protein revealing three distinct expression levels (low, medium, high) for both NDUFB8 and MTCO1 (Fig. 3C and G). The clustering results were validated using analysis of variance (ANOVA), which showed significant differences across the three clusters for NDUFB8/VDAC1 ratio and MTCO1/VDAC1 ratio, effectively categorising beta cells into groups with low, medium, and high expression. This clustering approach highlighted inter-donor heterogeneity, providing insights into mitochondrial function variability within the beta cell population (Supplementary Fig. 2D and E).

To account for the differences in total cell numbers across the cohorts, beta cell counts within each cluster were proportionally normalised by diabetes status. A LMM revealed significant differences in the expression patterns of beta cells based on diabetes status, confirmed by a Monte Carlo chi-squared test with Bonferroni correction across all clusters for both OXPHOS proteins (***P < 0.001). The overall cluster distribution differences showed medium effect sizes for both NDUFB8 (Cramer’s V = 0.202) and MTCO1 (Cramer’s V = 0.301), indicating meaningful shifts in mitochondrial protein expression patterns. Our analysis showed that in donors with type 2 diabetes, there was a significantly lower proportion of beta cells with high expression of complex I (NDUFB8) compared to donors without diabetes (high expression: type 2 diabetes: 1.77% vs without diabetes: 5.57%, respectively; ***P < 0.001), and a higher proportion with low expression (low expression: type 2 diabetes: 68.4% vs without diabetes: 46.0%, respectively; ***P < 0.001) (Fig. 3D). Conversely, for complex IV (MTCO1), a higher proportion of beta cells with high expression was observed in donors with type 2 diabetes compared to donors without diabetes (high expression: type 2 diabetes: 42.7% vs without diabetes: 15.5%, respectively; ***P < 0.001), while a lower proportion showed low expression (low expression: type 2 diabetes: 45.2% vs without diabetes: 60.7%, respectively; ***P < 0.001) (Fig. 3H). Our data suggest that beta cells from donors with type 2 diabetes tend toward low complex I expression and high complex IV expression patterns, though these proteins were analysed in separate staining panels.

## Discussion

This study provides key insights into mitochondrial protein expression in type 2 diabetes through single-cell resolution analysis. Our findings reveal distinct alterations in islet composition in type 2 diabetes, highlighting a novel OXPHOS protein expression profile in beta cells, characterised by reduced complex I and elevated complex IV protein expression.

Previous studies have identified significant differences in islet composition in type 2 diabetes. Our study corroborates these findings by observing alterations in islet composition when contrasting donors with type 2 diabetes and without diabetes (15, 17, 18). Using immunofluorescent labelling, consistent with prior immunohistochemical investigations of beta cell mass, we utilised our IsletAnalytics pipeline to identify a significant reduction in beta cell count in donors with type 2 diabetes compared to those without, aligning with the existing literature (26, 27, 28, 29). This resulted in a significant increase in the alpha-to-beta cell ratio, attributed to the metabolic strain induced by prolonged high blood glucose levels (30). Moreover, a substantial body of research has documented a loss of beta cell identity in donors with type 2 diabetes, characterised by an increased presence of trans-differentiated (bi-hormonal cells co-expressing glucagon and insulin) and de-differentiated (loss of beta cell transcription factors such as *Nkx6.1* and *Pdx1*) cells (24, 31, 32). In our cohort, we also identified a significantly greater number of bi-hormonal cells in donors with type 2 diabetes, despite their overall low abundance. This finding suggests a shift in beta cell identity, potentially reflecting an adaptive or maladaptive response to chronic metabolic stress.

To advance our understanding of OXPHOS protein expression in type 2 diabetes, we developed IsletAnalytics, a tailored single-cell segmentation image analysis pipeline built using established open-source software, to explore mitochondrial function in the context of type 2 diabetes. Our approach builds upon established techniques used in mitochondrial research, particularly the quadruple immunofluorescent labelling panel commonly designed to assess changes in OXPHOS complexes across diverse tissues and disease states (25, 33). In this study, we selected NDUFB8 and MTCO1 as markers of complex I and complex IV, respectively, owing to their roles as critical subunits, enabling precise assessment of mitochondrial protein expression (25, 33).

Given the complexity of analysing aged human tissue, the observed inter-donor heterogeneity in OXPHOS expression patterns reflects the expected biological variation in aged human tissue. Importantly, our LMM approach successfully captured consistent directional changes in complex I and IV expression across this heterogeneous population, underscoring the biological significance of these mitochondrial alterations in type 2 diabetes. In addition, we enhanced the analytical framework by integrating segmentation markers, which allowed us to identify beta cell subpopulations characterised by varying mitochondrial OXPHOS protein expression. Our findings align with previous single-cell omics studies highlighting substantial heterogeneity within endocrine cell populations, demonstrating the pipeline’s utility in capturing the complex cellular landscape of the islets (34, 35).

Before analysis, we standardised our approach by normalising OXPHOS protein expression to VDAC1, accounting for potential variable mitochondrial mass on a cell-by-cell basis. The recent literature suggests alterations in VDAC1 expression in type 2 diabetes, including its translocation toward the plasma membrane (36). However, our study found no evidence of differences in VDAC1 protein expression between donors with type 2 diabetes and age-matched donors without diabetes.

Our initial analysis revealed reduced complex I expression and increased complex IV expression in beta cells from older donors with type 2 diabetes compared to age-matched donors without diabetes. To further characterise heterogeneity within beta cell populations, we utilised a non-supervised cluster-based approach, revealing a significantly increased proportion of beta cells with low complex I expression and a significantly increased proportion exhibiting high complex IV expression in donors with type 2 diabetes. As mentioned in the introduction, previous studies have primarily focused on gene expression in human pancreatic islets, revealing significant downregulation of OXPHOS complexes (*NDUFA5*, *NDUFA10*, *COX11*, and *ATP6V1H*) in donors with type 2 diabetes (19). However, gene expression does not correlate with protein levels due to various regulatory mechanisms, making protein expression a more direct measure of functional gene products. The discordance between previously reported MTCO1 transcript reductions and our observed significant upregulation of MTCO1 protein suggests that transcript levels may not directly predict protein abundance in this context. This transcript-protein discordance has been documented in mitochondrial OXPHOS regulation, where respiratory chain complexes III and IV upregulation (125–262%) occurred without corresponding mRNA increases in ATP synthase deficiency, with mitochondrial mass controlled through porin normalisation and unchanged mtDNA content (37). The authors suggested post-transcriptional regulatory mechanisms, including translational control and protein stability, may account for such discordance. Similar regulatory processes may underlie the MTCO1 protein upregulation observed in type 2 diabetes islets in this study. Future investigations incorporating both transcriptomic and proteomic approaches will be necessary to characterise the specific mechanisms governing this discordance in the diabetic context.

Our results are consistent with findings from the *PolgA^mut/mut^* model, which exhibits significantly reduced complex I levels and increased complex IV levels in pancreatic beta cells at 44 weeks of age (14). Notably, this pattern diverges from other metabolically active tissues in the model, such as colonic epithelium and osteoblasts, where both complexes were significantly reduced (38, 39). This suggests that beta cells undergo distinct mitochondrial adaptations, potentially reflecting unique metabolic demands. Similar trends have been reported by Haythorne *et al.* (40) in a non-obese, eulipidaemic rodent model of beta cell dysfunction, which demonstrated coordinated downregulation of complex I at the transcript and protein levels. In contrast, complex IV transcripts were also diminished, but protein levels varied widely across individuals, suggesting post-transcriptional regulatory mechanisms governing OXPHOS protein expression under metabolic stress.

The distinct OXPHOS profile observed in our study reveals a previously unrecognised mitochondrial phenotype in type 2 diabetes, characterised by a significant increase in high complex IV protein expression in pancreatic beta cells. This finding mirrors observations by Ikonen *et al.* (41), who identified ‘hyperpositive’ MTCO1-stained skeletal muscle fibres in the *PolgA^mut/mut^* model, despite an overall reduction in complex IV protein levels. Interestingly, this phenotype was ameliorated by the expression of *Ciona intestinalis* alternative oxidase (AOX), which functions as a ROS attenuator. These findings support a mitochondrial model in which mitochondrial stress from various sources (including mtDNA mutations, environmental factors, or metabolic dysfunction) induces an adaptive response that may specifically contribute to beta cell dysfunction. This pattern, observed in both the *PolgA^mut/mut^* model and beta cells from individuals with type 2 diabetes, suggests that mitochondrial adaptations in beta cells are central to mitochondrial stress in this context. The emergence of ‘hyperpositive’ complex IV-expressing beta cells in our dataset may reflect a similar maladaptive response, reinforcing the rationale for targeting oxidative stress in therapeutic strategies aimed at preserving beta cell function in type 2 diabetes.

By focussing on an older human cohort, our study may partially reflect the impact of age-associated mtDNA mutations, which are known to accumulate with advancing age and contribute to oxidative stress and cellular dysfunction. We observed a distinct shift in OXPHOS protein expression within beta cells, marked by complex I reduction and elevated complex IV levels, consistent with chronic metabolic stress. The focus on an older cohort underscores the well-established association between advancing age and mtDNA mutation accumulation, a factor that may intensify oxidative stress and mitochondrial dysfunction (3), ultimately contributing to beta cell attrition in type 2 diabetes (4). While our study did not directly measure mutational load, Medini *et al.* (42) leveraged single-cell RNA sequencing to characterise beta cell subpopulations based on mtDNA expression and inferred mutational burden. Their identification of high-expression (HE) and low-expression (LE) subtypes suggests that HE cells may deploy adaptive mechanisms to counteract mtDNA damage and preserve function. However, their dataset did not distinguish by diabetes status or consider age, limiting its relevance to age-associated beta cell dysfunction. Our identification of beta cell subpopulations defined by divergent oxidative phosphorylation protein expression profiles raises the possibility that cumulative age-associated mitochondrial stress, including mtDNA mutations, may shape cellular resilience in human type 2 diabetes. Nonetheless, direct assessment of mtDNA variants will be necessary to validate this interpretation.

Here, we have identified beta cell subpopulations defined by OXPHOS protein expression, highlighting a critical intersection between ageing, mitochondrial adaptation, and disease progression. An outstanding question is whether the observed upregulation of complex IV represents a protective compensation or a maladaptive process that ultimately accelerates beta cell decline. Addressing this will require comparative analysis across age groups, to delineate age-related effects.

## Limitations

This study is limited by a modest sample size (*n* = 14) and the absence of autoantibody status, which prevents definitive exclusion of latent autoimmune diabetes in adults (LADA) from our type 2 diabetes cohort. Available clinical metadata includes HbA1c levels for four out of seven T2D donors (range: 44–93 mmol/mol) and diabetes duration for five out of seven T2D donors (range: <1–26 years). However, the incomplete coverage of HbA1c and duration data precludes statistical integration of these variables into our analytical models. In addition, diabetes medications are broadly categorised (insulin +/− medication, diet, or medication) without specific drug identification, preventing analysis of individual medication effects such as metformin’s influence on mitochondrial function. The four-channel imaging constraint also limited multiplexing capacity. While our immunofluorescent approach cannot directly assess OXPHOS functional activity, the VDAC1-normalised protein expression changes observed provide evidence of genuine per-mitochondrion alterations rather than simple organellar proliferation, establishing the essential phenotypic foundation for future functional investigations. Future studies should explore OXPHOS protein expression across additional endocrine cell types and in younger cohorts to assess age-related effects on pancreatic function.

## Conclusion

In this study, we developed a tailored image analysis pipeline using established open-source tools, machine learning, and automation to analyse immunofluorescently stained human pancreatic tissue at single-cell resolution. This enabled us to address specific questions about islet composition and mitochondrial protein expression in type 2 diabetes. We identified a distinct OXPHOS phenotype in beta cells from older donors with type 2 diabetes, marked by increased complex IV and decreased complex I expression. These findings suggest a potential metabolic adaptation in beta cells from donors with type 2 diabetes, offering new insight into mitochondrial dysfunction as a contributing factor to islet impairment.

## Supplementary materials



## Declaration of interest

The authors declare no conflict of interest that could be perceived as prejudicing the impartiality of the research reported.

## Funding

AM was supported by the John William Luccock and Ernest Jeffcock studentship. LG and AS were supported by CRUK (DRCPFA-Nov22/100001) and the Wellcome Centre for Mitochondrial Research (203105/Z/16/Z).

## Author contribution statement

CA, LG, and MW conceptualised the study. AM, XY, and AS contributed to data collection. AM was responsible for data analysis, processing, and quality control procedures. GM provided training and guidance for data analysis and processing. JS provided tissue for analysis, with additional support around sample inclusion. AM drafted the article, and AM, CA, LG, and MW were involved in draft revision. All authors reviewed the manuscript, and provided intellectual input, approved the final version of the manuscript, and accept responsibility for all aspects of the work, ensuring that any questions related to the accuracy or integrity of any part of the study are appropriately investigated and resolved. CA assumes overall responsibility for the integrity of the work.

## Data availability

The datasets generated from this study are available from the corresponding author upon reasonable request.
