# Supplementary material for: Single-cell analysis of oxidative phosphorylation protein expression in pancreatic islets in type 2 diabetes
Source: J Endocrinol. 2025 Oct 23;267(1):e250253. doi: 10.1530/JOE-25-0253 (PMC12910571; doi:10.1530/JOE-25-0253)
Supplement: Supplementary file 1 [file supplementary_materials.pdf]

Supplementary Table 1: Additional clinical characteristics of 14 human donors used in this study. Donors are categorised by diabetes status, with seven donors with type 2 diabetes and seven without diabetes. Data includes donor ID, age (in years), Body Mass Index (BMI, in kg/m^2), history of hypertension, history of cancer or malignancy, history of pulmonary disease, history of urinary tract infection(UTI), history of cardiac disease, history of liver disease and cold ischemia time (hours).

Donor ID	Age (years)	BMI (kg/m^2)	History of hypertension	History of Cancer or Malignancy	History of Pulmonary Disease	History of UTI	History of Cardiac Disease	History of Liver Disease	Cold Ischemia time (Hours)
Mean ± SEM (Type 2 Diabetes)	65.43 ± 1.36	32.13 ± 1.82							
Mean ± SEM (Without Diabetes)	67.0 ± 2.04	29.80 ± 1.01							
P-Value	0.533	0.285							
PT0267_0020	70	34.9	Y	N	N	N	N	N	5.83
PT0267_0026	63	32.8	N	N	N	Y	N	N	4.42
PT0267_0051	71	28.6	Y	Y	N	N	N	N	5.8
PT0267_0081	63	33.3	Y	N	N	N	N	N	21.65
PT0267_0086	64	27.5	Y	N	N	N	N	N	10.07
PT0267_0090	65	40.6	Y	N	Y	N	N	N	21.8

PT0267_0095	62	27.2	Y	N	Y	N	Y	N	15.5
PT0267_0021	63	31.6	N	N	N	N	N	N	7
PT0267_0022	63	26.9	N	N	N	N	N	N	21.1
PT0267_0030	62	28.7	Y	N	N	N	N	N	21.25
PT0267_0032	74	30.5	N	N	N	N	N	N	11.72
PT0267_0042	71	26.3	Y	N	N	N	N	N	2.7
PT0267_0054	63	30.8	N	N	N	Y	N	N	6.77
PT0267_0058	73	33.8	Y	N	N	N	N	N	10.8

IsletAnalytics Analysis Tool

Presented below is a detailed overview of the semi-automated machine learning-based image analysis pipeline (**Figure 1**).

1 Installation of required software.

ImageJ, Ilastik and CellProfiler are all open-access software that is compatible for use on macOS and Windows.

2 Convert image file format.

The vendor-specific image format generated by the Zeiss Laser Scanning Microscope 800 is .czi, which is incompatible with downstream analysis in Ilastik and CellProfiler. To address this limitation, a comprehensive ImageJ macro was developed to perform automated batch conversion and image preparation for subsequent analysis.

The macro workflow commences with user-defined directory selection, prompting specification of the home folder (containing all project subdirectories) and the raw image folder (housing .czi files). Upon execution, the macro automatically generates a standardised directory structure, creating distinct folders for segmented islets, cropped individual islets, scaled images optimised for Ilastik processing, and Ilastik probability map outputs.

File conversion is achieved through Bio-Formats Importer integration, with each .czi file opened using autoscaling parameters and default colour mode settings. The macro systematically processes images by scaling to 50% of original dimensions to optimise computational efficiency during subsequent Ilastik analysis, while preserving image quality for accurate segmentation. Converted images are saved in .tiff format with standardised naming conventions to facilitate automated batch processing.

The macro incorporates direct Ilastik integration through automated pixel classification prediction calls. Using a pre-trained project file (.ilp), scaled images are processed through the established random forest classifier to generate probability maps for islet segmentation. Output probability maps undergo 8-bit conversion for compatibility with downstream CellProfiler analysis. Following Ilastik processing, images are rescaled to original dimensions using bilinear interpolation to maintain spatial accuracy for quantitative measurements.

Quality control measures are embedded throughout the workflow, including file existence verification to prevent redundant processing, automated skip functionality for previously processed images, and systematic error handling for corrupted or incompatible file formats. The macro maintains processing logs and provides real-time status updates to monitor workflow progression across large datasets.

This automated approach ensures reproducible file conversion and preparation while minimising manual intervention and potential human error. The modular design permits adaptation for alternative microscope systems and file formats through modification of Bio-Formats import parameters and scaling factors, providing flexibility for diverse experimental configurations. The complete ImageJ macro code and implementation details are available at: https://github.com/amullins2/HumanIsletMLPipeline_2024

3 Generate image stacks.

Following file conversion (Stage 2), the generated .tiff image formats are converted to a stack .tiff format, splitting the channels, applicable for further analysis e.g., Ilastik and CellProfiler. In this pipeline, the generation of four stack images per islet is produced, two-image stacks are required in Ilastik, and four-image stacks are required in CellProfiler. This step is also adaptable based on the design of the study.

4 Ilastik Iterative Training.

Ilastik, a machine learning tool for pixel classification, was utilised to segment complete islets in proximity within the islet composition staining panel, facilitating individual analysis of each islet. To initiate training in Ilastik, a new pixel classification project is created, and a diverse set of four images from different donors is imported to ensure the representation of staining variability across donors.

The selection of features for classification is crucial. While the software recommends utilising as many features as computationally feasible, it's important to note that the addition of features requires higher random-access memory (RAM). Thus, on systems with limited RAM, excessive features may slow down Ilastiks performance.

For the pixel classification process, two classifications, Islets and Background, are chosen for segmenting complete islets. It is advisable to use small brush sizes and label sparingly. Ilastik operates as an observational trainer, allowing for additional annotations without the need for larger brush sizes. Moreover, using larger brush sizes can lead to software malfunctions and disrupt the training process.

In the classification window, located in the lower left-hand corner, channels for the stacked images can be modified for optimal training efficiency. Initially, outlining the edges of islets and sparingly drawing lines in the centre is recommended, followed by using the background pen to draw sparing lines in background areas. Selecting 'Live Update' allows for real-time monitoring of uncertainty and trained segmentation progress. Pixel classification is considered complete when the uncertainty appears almost transparent.

Once the random forest classifier is trained, the remaining images can be batch-processed.

5 Nuclei Segmentation in the Islet Composition Panel with Stardist.

Stardist, an ImageJ/FIJI plug-in, is specifically tailored for automated nuclei detection. While CellProfiler was initially considered for nuclei segmentation, comparative analysis between manual segmentation, CellProfiler, and Stardist revealed that Stardist outperformed in nuclei segmentation, thus providing optimal results.

It is noteworthy that all steps of the image analysis pipeline mentioned above can be executed within an automated ImageJ/FIJI macro. However, it is important to highlight that in mitochondrial function staining panels, the focus is only beta cell function within pancreatic islets, where the nuclei marker DAPI is absent. Consequently, the utilisation of Ilastik and Stardist is unnecessary in such scenarios.

6 Single-Cell Segmentation and Quantification of Single-Cell Features in CellProfiler.

For segmenting islets into single cells, a CellProfiler pipeline was implemented, with variations tailored for the islet composition and mitochondrial function staining panels, depending on the specific segmentation stains utilised.

In the analysis of the islet composition staining panel, after nuclei segmentation in Stardist, islets are further divided into their respective channels: nuclei (DAPI), cell membrane (E-cadherin), glucagon (alpha cells), and insulin (beta cells) using an automated ImageJ/FIJI macro. Subsequently, these split channel images are processed in CellProfiler, where various image processing modules are applied to enhance output images for analysis. Modules such as "ReScaleIntensity" optimise intensity for the E-cadherin segmentation marker, while "Smooth" removes any artefacts from the E-cadherin segmentation marker stain and enhances signal continuity by filling gaps within the segmented regions. The 'IdentifyPrimaryObjects' module is then applied to recognise segmented nuclei images from the Stardist output. Following this, the 'IdentifySecondaryObjects' module is utilised with the nuclei as primary objects and the cell membrane segmentation as secondary objects, employing a three-class Otsu thresholding method (threshold range: 0.02-0.4) with propagation. This approach allows accurate identification of individual cells based on the segmented nuclei and membrane signals. Glucagon and insulin intensities within individual cells are measured using the "MeasureImageIntensity" and "MeasureObjectSizeShape" modules, with the output saved to a .csv file via the "ExportToSpreadsheet" module.

In the analysis of the mitochondrial staining panels, the workflow in CellProfiler differs due to the presence of only one segmentation marker, a limitation imposed by the availability of only four fluorophore channels. Here, the 'IdentifyPrimaryObjects' module is used to recognise the cell membrane (E-cadherin) marker images, eliminating the need for the 'IdentifySecondaryObjects' module. Similar to the islet composition staining panel, cell membrane segmentation utilises three-class Otsu

thresholding within a defined range of 0.07-0.1. Measurement of complex I, complex IV, mitochondrial mass, and insulin intensities within individual cells is then performed using the "MeasureImageIntensity" and "MeasureObjectSizeShape" modules, with the output saved to a .csv file through the "ExportToSpreadsheet" module."

7 CellProfiler Neighbourhood Analysis within Islet Composition Staining Panel.

To compute inter-islet differences, neighborhood analysis was also performed on the islet composition staining panel within CellProfiler (Figure 2). The “ClassifyObjects” module is used to assign alpha and beta cell donor-based threshold values, to establish the two cell types within islets. The module “MeasureObjectNeighbours” then quantifies for each donor the number of alpha-alpha, beta-beta and beta-alpha inter-islet cell connection proportions.

Supplementary Table 2 – Primary Antibodies used in this study.

Staining Panel	Primary Antibody	Species raised in	Clonality	Isotype	Confirmed Specificity	Dilution	Reference Number
NDUFB8 (Complex I) Primary Antibodies	NDUFB8	Mouse	Monoclonal	IgG1	Mouse, Rat, Cow, Human, Pig	1:200	Abcam ab110242
	VDAC1	Mouse	Monoclonal	IgG2b	Mouse, Rat, Cow, Human	1:100	Abcam ab14734
	Insulin	Guinea-Pig	Polyclonal	IgG	Human, Mouse, Rat	1:5	DAKOIR00261-2
	E-cadherin	Rabbit	Monoclonal	IgG	Human, Mouse	1:500	Cell Signaling 24E10
MTCO1 (Complex I) Primary Antibodies	MTCO1	Mouse	Monoclonal	IgG2a	Human, Mouse, Rat	1:200	Abcam ab14705
	VDAC1	Mouse	Monoclonal	IgG2b	Mouse, Rat, Cow, Human	1:100	Abcam ab14734
	Insulin	Guinea-Pig	Polyclonal	IgG	Human, Mouse, Rat	1:5	DAKOIR00261-2
	E-cadherin	Rabbit	Monoclonal	IgG	Human, Mouse	1:500	Cell Signaling 24E10
Islet Composition Primary Antibodies	Insulin	Guinea-pig	Polyclonal	IgG	Human, Mouse, Rat	1:5	DAKOIR00261-2
	Glucagon	Mouse	Monoclonal	IgG1	Rat, Pig, Guinea-pig, Mouse, Canine, Human, Rabbit	1:100	Sigma G2654
	E-cadherin	Rabbit	Monoclonal	IgG	Human, Mouse	1:500	Cell Signaling 24E10

Supplementary Table 3 – Secondary Antibodies used in this study.

Staining Panel	Species raised in and isotope	Antibody against	Conjugation	Dilution	Reference Number	Corresponding Primary Antibody
NDUFB8 (Complex I) Secondary Antibodies	Mouse IgG1	Goat	Biotin	1:200	Thermo Fisher Scientific A10519	NDUFB8
			Strepavidin Alexa 647	1:100	Thermo Fisher Scientific S32357	
	Mouse IgG2b	Goat	Alexa Fluor 488	1:500	Thermo Fisher Scientific A21141	VDAC1
	Guinea Pig IgG	Goat	Alexa Fluor 405	1:200	Abcam ab175678	Insulin
	Rabbit IgG	Goat	Alexa Fluor 546	1:500	Thermo Fisher Scientific A21085	E-cadherin
MTCO1 (Complex I) Secondary Antibodies	Mouse IgG2a	Goat	Alexa Fluor 647	1:200	Thermo Fisher Scientific A21241	MTCO1
	Mouse IgG2b	Goat	Alexa Fluor 488	1:500	Thermo Fisher Scientific A21141	VDAC1
	Guinea Pig IgG	Goat	Alexa Fluor 405	1:200	Abcam ab175678	Insulin
	Rabbit IgG	Goat	Alexa Fluor 546	1:500	Thermo Fisher Scientific A21085	E-cadherin
Islet Composition Secondary Antibodies	Guinea Pig IgG	Goat	Alexa Fluor 647	1:250	Thermo Fisher Scientific A21450	Insulin
	Glucagon	Donkey	Alexa Fluor 568	1:250	Thermo Fisher Scientific A10037	Glucagon
	Rabbit IgG	Goat	Alexa Fluor 488	1:500	Thermo Fisher Scientific A11008	E-cadherin

Supplementary Figure 1

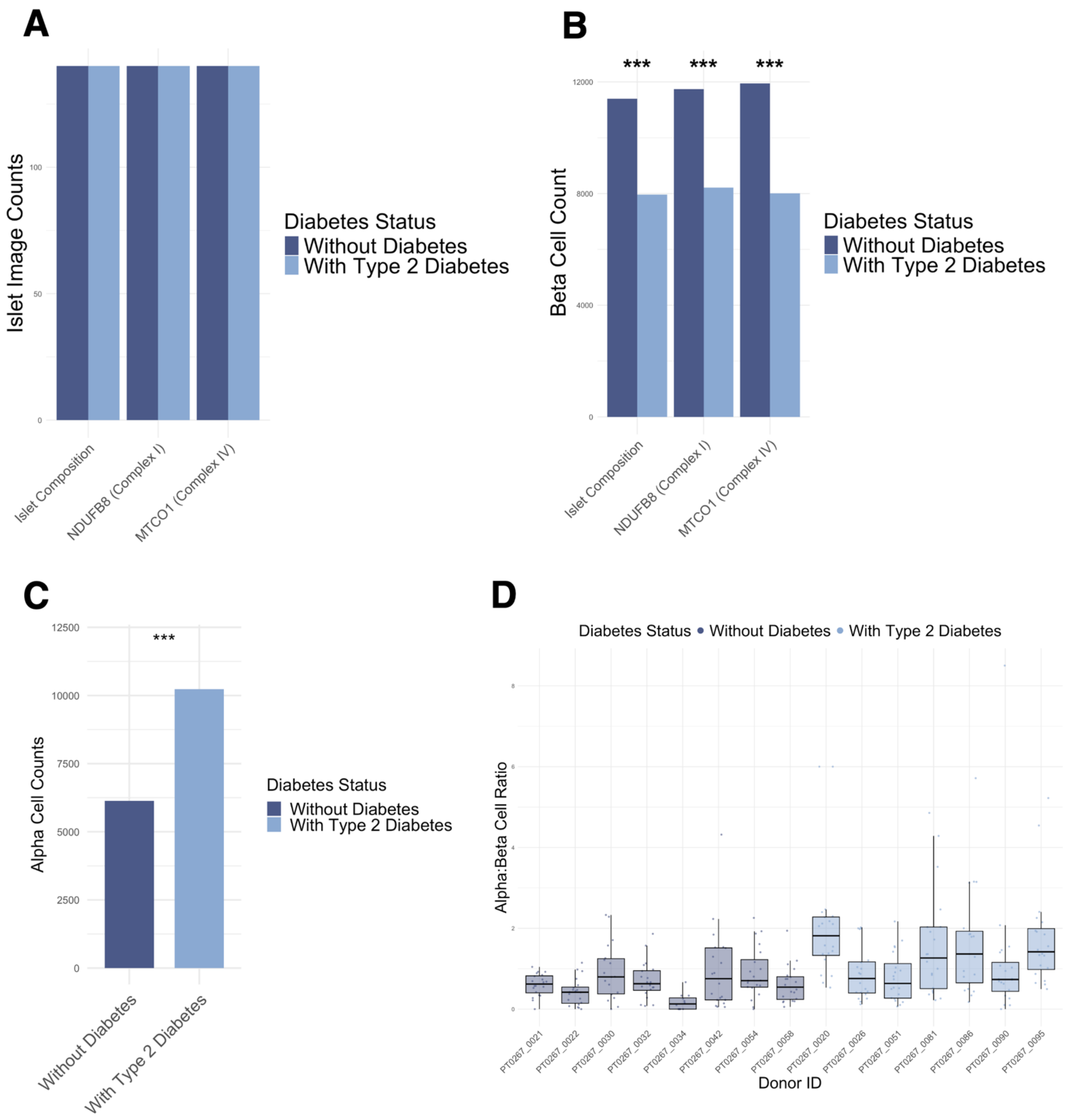

Supplementary Figure 1 Figure Legend:

A) Bar graph showing the number of islet images analysed across three datasets: Islet Composition, NDUFB8, and MTCO1. Counts are stratified by diabetes status, with donors without diabetes represented in dark blue and those with type 2 diabetes in light blue. Comparable numbers of islet images were analysed across all datasets. **B**) Bar graph of beta cell counts for the same three datasets. In each case, donors without diabetes exhibited significantly higher beta cell counts than those with type 2 diabetes (*$***P < 0.001$*), highlighting a consistent reduction in beta cells in type 2 diabetes. **C**) Bar graph showing alpha cell counts from the islet composition staining panel. Alpha cell counts were significantly higher in donors with type 2 diabetes compared to those without diabetes (*$***P < 0.001$*). **D**) Box plot showing the alpha: beta cell ratio by donor ID, stratified by diabetes status. Each group is represented by a box plot displaying the median, quartiles, and outliers, with individual islet data points overlaid as jittered dots. Statistical comparisons were performed using a linear mixed model with post-hoc Monte Carlo Bonferroni correction. Significance: $*P < 0.05$; $**P < 0.01$; $***P < 0.001$.

Supplementary Figure 2

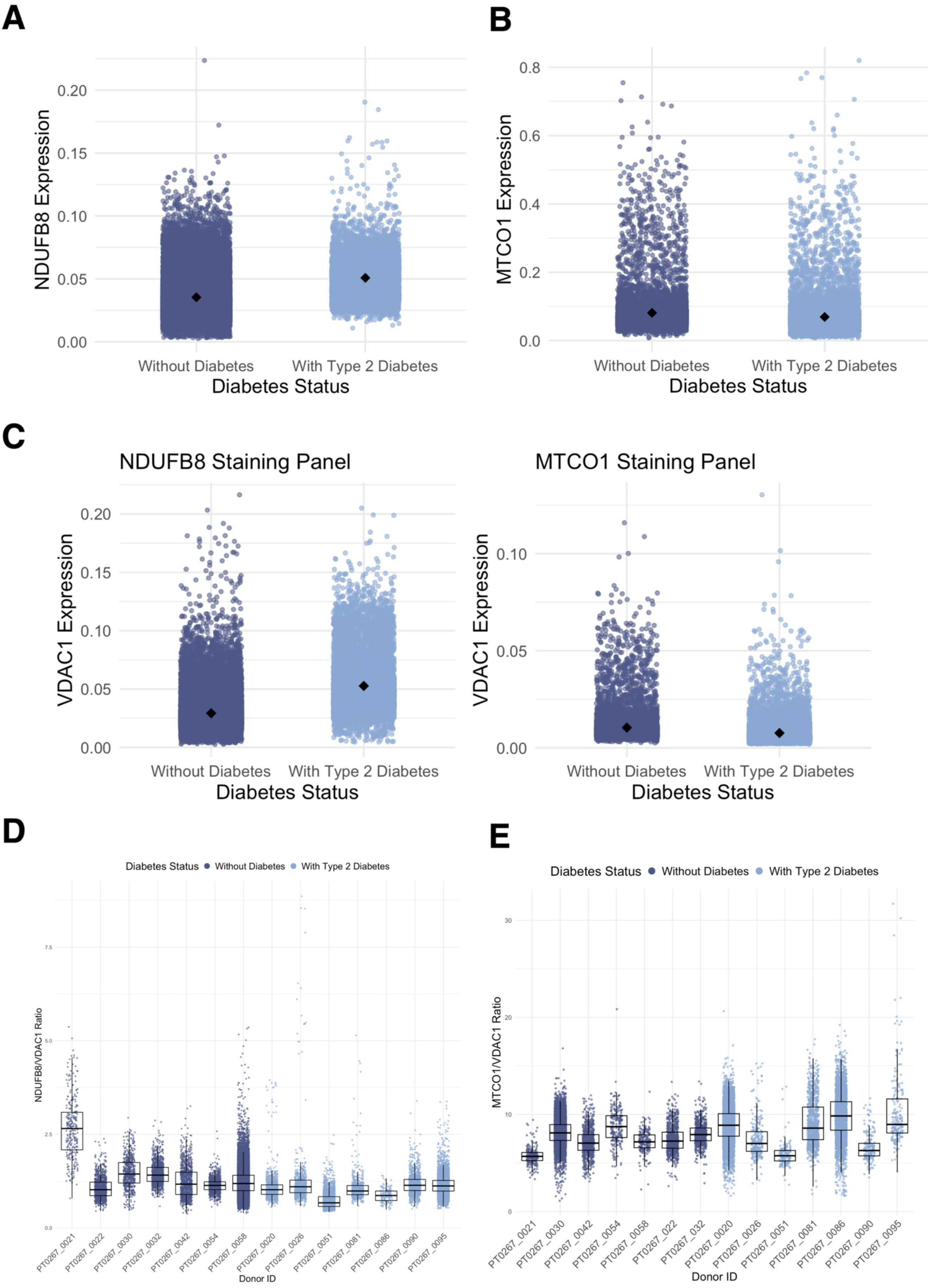

Supplementary Figure 2 Figure Legend:

A & B) Jitter plots illustrate the distribution of NDUFB8 (A) and MTCO1 (B) expression in individual beta cells, stratified by diabetes status. Each plot represents data from *n = 14* donors (seven without diabetes and seven with type 2 diabetes), with individual beta cells plotted against diabetes status (x-axis). Black diamonds indicate the group mean. **C**) Jitter plots showing VDAC1 expression individual beta cells from the same donors, based on the NDUFB8 and MTCO1 staining panels, respectively. As in panels A and B, black diamonds represent mean expression per group. Statistical analysis using a linear mixed model showed no significant differences between groups ($P = 0.461$). **D**) NDUFB8/VDAC1 ratio for individual beta cells, grouped by donor ID on the x-axis. Jittered points represent individual cell values, coloured by diabetes status (left = without diabetes; right = with type 2 diabetes). Box plots summarise the distribution by diabetes group, showing the media, interquartile range, and outliers. **E**) MTCO1/VDAC1 ratio per beta cell, displayed similarly to panel D. Data are grouped by donor ID, with jittered points coloured by diabetes status and overlaid on box plots illustrating group distributions. Significance: *$P < 0.05$; **$P < 0.01$; ***$P < 0.001$.
